# Projected Climate and Hydroregime Variability Constrain Ephemeral Wetland-Dependent Amphibian Populations in Simulations of Southern Toads

**DOI:** 10.3390/ecologies3020018

**Published:** 2022-06-17

**Authors:** Jill A. Awkerman, Cathryn H. Greenberg

**Affiliations:** 1 Gulf Ecosystem Measurement and Modeling Division, US EPA, One Sabine Island Drive, Gulf Breeze, FL 32561, USA; 2 USDA Forest Service, Southern Research Station, Bent Creek Experimental Forest, 1577 Brevard Rd., Asheville, NC 28806, USA

**Keywords:** amphibian, climate, pesticide, hydroregime, ephemeral wetland, population model, risk assessment, southern toad

## Abstract

Amphibian populations are threatened globally by stressors, including diminishing availability of suitable wetland breeding sites, altered hydroregimes driven by changing weather patterns, and exposure to contaminants. Ecological risk assessment should encompass spatial and temporal scales that capture influential ecological processes and demographic responses. Following the PopGUIDE framework of population model development for risk assessment, we used matrix population models, in conjunction with existing hydroregime predictions, under a climate change scenario to evaluate the effects of environmental stochasticity and aquatic pesticide exposure on amphibians that are dependent on ephemeral wetlands. Using southern toads (*Anaxyrus terrestris*) as an example, we simulated population dynamics with breeding success dependent on hydroregime suitability. Years were defined as optimal, marginal, or insufficient for successful toad recruitment, based on the duration of their potential breeding season and rate of larval development to metamorphosis. We simulated both probabilistic and chronologically specific population projections, including variable annual fecundity, based on hydroregime suitability and reduced larval survival from carbaryl exposure. In our simulations, populations were more negatively impacted by prolonged drought, and consequently multiple sequential years of reproductive failure, than by aquatic pesticide exposure. These results highlight the necessity of reliable climate projections to accurately represent the effects of altered hydroregimes on amphibian populations. Risk assessment approaches could be improved with flexible modifications that allow inclusion of various extrinsic stressors and identification of demographic and ecological vulnerabilities when precise data are lacking.

## 1. Introduction

Given the many drivers of amphibian decline globally, the difficulty of assessing population-level risk is complicated by the heterogeneous distribution of pathogens, parasites, and pesticides, as well as a diversity in life history traits among amphibians [1,2]. Complex biphasic life cycles and hydroregime requirements differ among amphibian species, highlighting the need for detailed demographic and ecological data that properly represent species-specific population dynamics, prior to estimating risk of extrinsic stressors that vary within a species’ range. Realistic representations of reproductive success must incorporate environmental stochasticity and long-term variability in hydroregime (duration, timing, frequency, and depth of water in wetlands), particularly for species dependent on ephemeral wetlands for successful recruitment (larval development through metamorphosis). Variable reproductive success and recruitment as a result of environmental stochasticity is likely to impact population projections, emphasizing the importance of incorporating temporal resolution consistent with typical population fluctuations. Determining an ecologically realistic representation of population dynamics, prior to evaluating the effects of stressors, is important for appropriate context relative to baseline conditions.

Reproductive success for wetland-breeding amphibians is often linked to precipitation patterns and wetland hydroregime [3]. Drought or flooding can determine whether a local population reproduces in a given year, and the increasing frequency of extreme weather events, associated with a changing climate, is further motivation to examine long-term impacts of altered weather patterns on amphibian reproductive success [4,5]. Effects of climate-related changes on successful recruitment are largely dependent on the reproductive strategy of individual species, namely the breeding through metamorphosis period (BMP), that encompasses the season and length of the breeding phase and time required for completion of larval development to metamorphosis [6]. Thus, identification of meaningful life history traits could offer a first step when inferring potential impacts of climate-driven changes to hydroregime on different wetland-breeding amphibian species [6].

Screening-level identification of traits associated with population vulnerability among species is valuable as an initial step given the extensive data requirements to effectively measure empirical demographic rates under variable conditions [7–10]. Identifying intrinsic species vulnerability offers a starting point to effectively mitigate extrinsic stressors contributing to population decline. The benefit of population-level ecological risk assessment is clear [11,12], but sufficient monitoring data for such perspectives come at a cost of years to decades [12]. Comprehensive risk assessment necessitates evaluation of potential stressors, such as climate change and pesticides, as well as demographic and ecological traits associated with population vulnerability [13]. Previous population model simulations of anuran species with similar life histories suggest that unsuitable hydroregimes relative to their BMP reduces, or eliminates, breeding opportunity and larval survival, potentially resulting in complete reproductive failure and local population decline [14,15]. Extrinsic stressors are likely to further exacerbate threats to population viability.

Amphibian species reliant on ephemeral wetlands are particularly susceptible to variation in hydroregime [6,16,17]. These temporary waterbodies vary in size, depth, timing, and duration of inundation, depending on precipitation patterns, underlying substrate, and groundwater levels. Wetland hydroperiods must coincide with the timing and duration of the BMP for species breeding at these wetlands to allow for potentially successful breeding and metamorphosis to occur in any given year. Environmental factors, such as the timing and amount of precipitation, and pre-existing groundwater levels can affect the overlap between BMP and suitable hydroregimes in wetlands, potentially impacting population dynamics [6,16,17]. Additional negative impacts on larval survival or development, such as those caused by pesticide exposure, could contribute to population decline.

Carbaryl is a carbamate insecticide that causes cholinesterase inhibition and is frequently used in orchards and vegetable crops. It is highly toxic to aquatic invertebrates and can affect amphibian development and survival at environmentally relevant concentrations [18]. Historically, carbaryl was fairly ubiquitous, found in approximately half of urban streams, and the second most frequently detected pesticide in water [19]. In other studies, interpretation of long-term effects on amphibian populations is complicated by interactions of indirect effects, density effects, and predation, along with differences in effects between species [20,21].

In southern toads (*Anaxyrus terrestris*), carbaryl exposure (3.5 mg L^−1^) resulted in reduced larval survival [22]. Southern toads are a common amphibian species breeding in ephemeral wetlands of the southeastern Coastal Plain and are a species of minimal conservation concern, suggesting minimal intrinsic vulnerability. Their long breeding season and rapid larval development offers greater opportunity for potentially successful reproduction, even in wetlands with intermittent hydroperiods within their BMP. Our focus on southern toads offers a case study in population modelling for a species with demographic traits that are less vulnerable to reduced hydroperiod.

Population-level ecological risk assessment has long been lauded for the ability to incorporate demographic, ecological, and extrinsic sources of variance in vital rates for multiple taxonomic groups, including amphibians [12], thereby providing a more meaningful evaluation of population viability in conservation biology and risk assessment [23–26]. However, the lack of a standardized approach yielding reproducible, defensible decisions has hindered implementation of population modeling in risk assessment. In response, recent papers have focused on better defining the risk assessment framework and decision steps to guide population modeling techniques [27,28], including life history nuances and stressors that are particularly relevant to different taxonomic groups (e.g., [13]).

Here we use the stepwise population-level ecological risk assessment guidance of PopGUIDE [29] to provide a representative population model for the southern toad under different hydroregime forecasts that were developed by Greenberg et al. [30] for eight ephemeral wetlands over a 50-year period (2012–2060), using the CSIRO (Commonwealth Scientific and Industrial Research Organization) Mk3.5 General Circulation Model (GCM) under an A1B emissions scenario. The GCM represents one relatively moderate set of assumptions with regard to future environmental, political, and technological changes. Specifically, the A1B scenario assumes a modest global population size and a balance between fossil fuels and alternative energy sources. An updated version of this model is available (CSIRO Mk3.6 GCM) that differs in its radiation scheme and interactive treatment of aerosol but remains unchanged with regard to the sea-ice, ocean, and soil-canopy models [31]. Since our focus was on the population (not hydrological) model development, we elected to retain the existing hydroregime suitability classifications for wetlands based on the Mk3.5 projections used by Greenberg et al. [30], rather than develop updated hydrodynamic models. Further, CSIRO-Mk3.5 was determined to perform well [32], and updated climate models did not substantially differ in predictions of breeding habitat suitability for amphibians in California [33].

Some of the eight focal wetlands in Greenberg et al. [30] had similar patterns of predicted hydroregime suitability for southern toads. Therefore, we selected a subset (four) of those wetlands representing different proportions of suitable hydroregime years, in order to illustrate potentially contrasting outcomes for our population simulations. We classified hydroregimes within each year and wetland as optimally, marginally, or insufficiently suitable for southern toad reproductive success, based on known BMPs [6,30], to simulate long-term population dynamics. The decision tree framework described in Raimondo et al. [29] determines the risk assessment category (realistic, general, precise, etc.) that best describes the desired model strengths, and model development follows the phases outlined by their methodology to define model objectives. Our objectives were: (1) model southern toad population dynamics, based on temporal and spatial variability in projected hydroregime suitability, (2) additionally simulate hypothetical exposure to the pesticide carbaryl during the larval stage and identify its impact on long-term population dynamics, and (3) determine the utility of a probabilistic projection of variable reproductive success to evaluate long-term population dynamics when hydroregime suitability data or projections are lacking. Examining population fluctuations associated with environmental variability informs the temporal and spatial scale that adequately captures typical population dynamics and impacts of extrinsic stressors within an ecologically relevant context.

## 2. Materials and Methods

**Figure 1.**
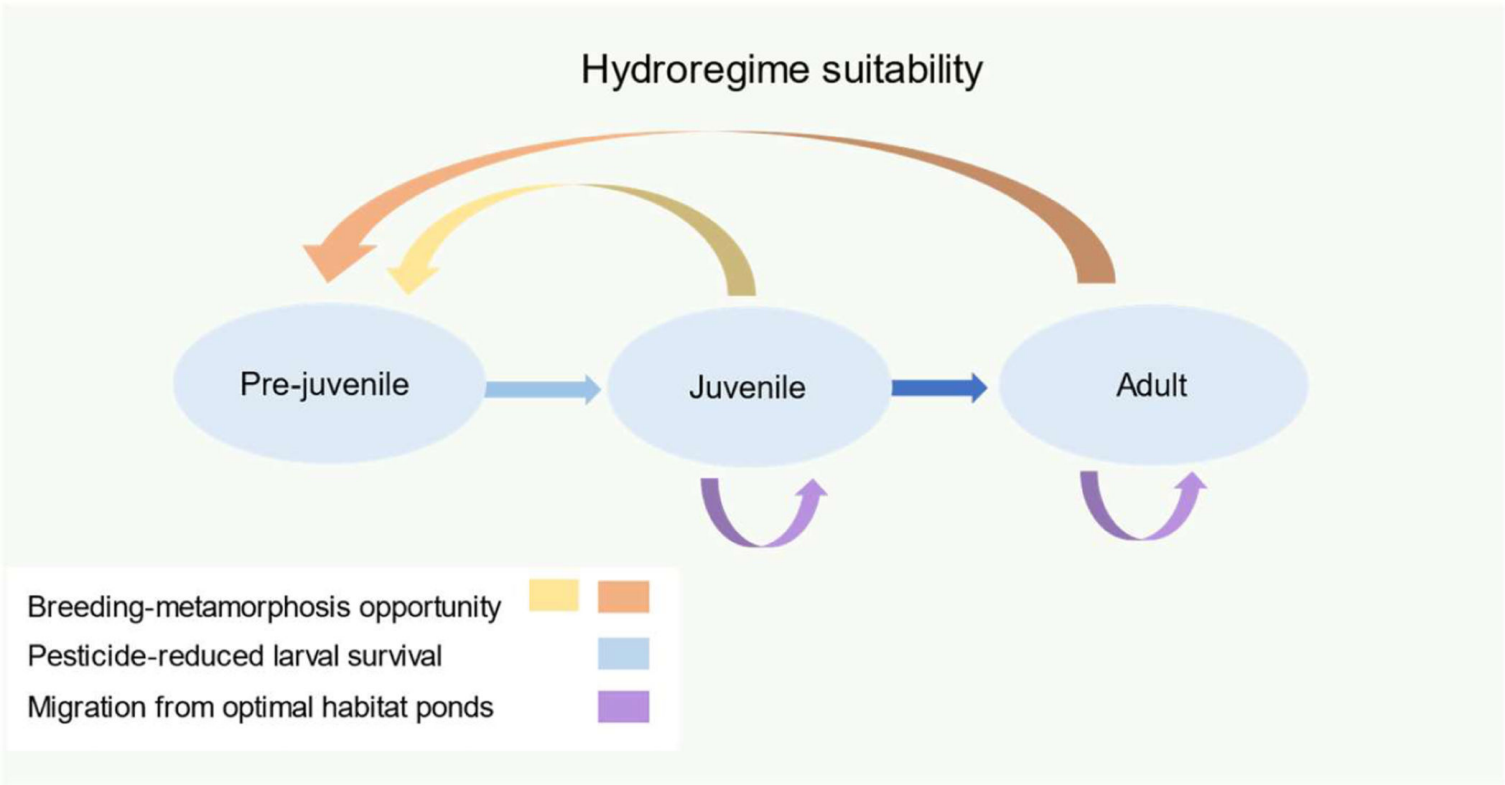
Life history representation demonstrating potential adjustments for hydroregime suitability, including interannual breeding-through-metamorphosis opportunity for both adults and juveniles, reductions in larval survival in response to aquatic pesticide exposure, and possible migration from nearby ponds with increasing subpopulations.

Steps in the PopGUIDE model development process are described elsewhere [29]; briefly, we identified model objectives (Phase I), compiled data (Phase II), followed decision steps (Phase III), developed a conceptual model (Phase IV; Figure 1), and evaluated the model (Phase V).

### 2.1. Identification of Assessment Type and Model Objectives (Phase I)

Our primary objective in model development was to evaluate the consequence of environmental stochasticity, specifically the projected variance in the timing and amount of precipitation on hydroregime and consequent effects on interannual and inter-wetland breeding success. The addition of theoretical exposure during the aquatic phase to the pesticide carbaryl was a secondary objective to evaluate its potential effects on recruitment in the context of spatial and temporal variability of suitable hydroregimes.

In our model we used the southern toad, a common anuran in the southeastern Coastal Plain, as an example, but other species could be modeled using species-specific demographic and habitat details. Since our focus on environmental stochasticity, and portraying pesticide exposure in that ecologically realistic context, was not motivated by interest in a specific species or location, our model is described as general-realistic, according to PopGUIDE decision tree, suggesting a compromise in model precision (*sensu* [34]). Model objectives initially addressed theoretical impacts of environmental stochasticity (hydroregimes) alone on southern toad population growth without confounding factors, such as pesticides, pathogens, or the limited spatial distribution constraints of an endangered species. We included aquatic pesticide exposure effects in subsequent projections.

### 2.2. Data Compilation (Phase II)

The southern toad has an International Union for Conservation of Nature status of “least concern”. It is ubiquitous in the southeastern United States Coastal Plain and has a relatively short larval development period and lifespan (Table 1), and life history traits that suggest minimal inherent population vulnerability [15].

We based annual fecundity estimates on existing hydroregime projections for isolated, ephemeral, groundwater-driven sinkhole wetlands located in longleaf pine-wiregrass sandhills in the Ocala National Forest, Marion County Florida (see [30] for more detail). Greenberg et al. [30] used 17 years (1994–2011) of rainfall and wetland depth data from their eight study wetlands to develop a predictive model for hydroregimes. They then used their model with downscaled climate data from the CSIRO Mk3.5 GCM under an A1B emissions scenario to forecast weekly water depths and hydroperiods over a 50-year period (2012–2060). Based on these forecasted hydroregimes they identified years with hydroregimes conducive for successful breeding and metamorphosis for five anuran species, including southern toads. Hydroregimes for each pond and year were classified as optimal, marginal, or insufficient (identified here as OHY, MHY, and IHY, respectively) based on the duration of water (>1 cm) in wetlands in relation to BMP for each species. For southern toads they defined OHY as water continuously in a wetland for the duration of the maximum BMP (February week 2 through August week 2); MHY as water continuously in a wetland for the minimum time required for development of a single cohort within the BMP (any six weeks between March week 1 and August week 2), and IHY as less than six continuous weeks of water at any time during the BMP, which would result in tadpole desiccation before metamorphosis and, therefore, reproductive failure [30]. Greenberg et al. [30] examined how forecasted water depths and hydroperiods might affect reproductive success of southern toads and other anurans but did not model population dynamics. Vital rate estimates and assumptions used in southern toad population models are described in Awkerman and Raimondo [15] and summarized below. Briefly, we compiled reproductive and survival data for southern toads, or taxonomically and ecologically similar species, from a number of field and mesocosm studies [6,14,20,30,35,36] to estimate stage-specific annual survival, fecundity, and transition rates.

### 2.3. Model Decision Steps (Phase III)

PopGUIDE model parameterization is categorized in five model steps identified herein and described in detail elsewhere [27]. Parameterization of model and assumptions are discussed in Phase IV (Conceptual Model). Life history representation for southern toads (step 1; Figure 1) was based on a previously developed female-only matrix model with an annual time step and post-breeding census, comprising three life stages: pre-juvenile (comprising the entirety of aquatic embryolarval development), juvenile (terrestrial life stage prior to reproductive maturity), and adult [15]. Fecundity estimates were derived from the assumed hydroregime-determined proportion of successful breeders in addition to estimated demographic rates used in previous population models [14,15]), including proportion of surviving breeders, clutch size, embryo survival, and larval survival. Metamorphs transitioned into the juvenile stage for an additional two years and were considered breeding adults in the third year. Focal elements of organism-level processes (step 2), population and spatial factors (step 3), external factors (step 4), and pesticide exposure and effects characteristics (step 5) are summarized in Table 1. Briefly, organism-level processes were estimates of vital rates (i.e., survival, fecundity) compiled from previous field studies [6,14,22,30]. Step 3 summarized details of the populations and spatial factors relevant to the wetlands used in our models, with initial population size based on wetland area [35] and experimental enclosure density estimates for similar species [36]. Terrestrial density dependence was used to limit population growth, based on estimated overwintering densities in enclosures of American toads (*A. americanus*) [36]). Initial population sizes merely provided a standardized and realistic starting point and were not intended to estimate populations precisely. Likewise, final population projections were meant to provide context on population fluctuations, rather than abundance estimates or location-specific management criteria. The external factors (step 4) identified for initial simulations focused primarily on environmental stochasticity-associated hydroregime variability and did not include predation, pathogens, competition, or indirect effects. Spatial (wetland) and temporal (year) specific differences in anticipated hydroregime were represented as a differential proportion of breeders, depending on the duration of water (>1 cm depth) present relevant to the BMP of the southern toad.

Four distinct patterns in hydroregime suitability for southern toads were apparent in the four wetlands we selected for our simulations (wetlands 2, 3, 5, and 7 in Greenberg et al.’s study [30]. These hydroregime patterns resulted in IHY for approximately 20, 40, 50, and 80% of years modeled. In our models, we used schedules of projected hydroregime suitability categories (OHY, MHY, ISY) to represent these four patterns and varied the fecundity estimates (number of female metamorphs produced per female in that year) accordingly for population projections (2012–2060). Assuming that hydroregime (OHY, MHY, or IHY) influenced the proportion of successful annual breeders (0.67, 0.33, or 0, respectively), resulted in approximate breeding frequencies similar to interannual estimates reported elsewhere for southern toads (0.3; [14]). Pesticide effects (step 5) were added to these projections in a separate set of population simulations. Exposure to carbaryl during aquatic life stages was included as a reduction in larval survival during years with suitable hydroregimes during the BMP (OHY or MHY classifications).

### 2.4. Conceptual Model (Phase IV)

We modified matrix models previously developed for comparative sensitivity analysis [15] using R [37]. These provided a simple tool for projection under different sets of assumptions regarding potential effects of hydroregime variability on breeding success, larval survival to metamorphosis, and dispersal or migration of individuals during the terrestrial phase (Figure 1).

In projected simulations we assessed the influence of spatial and temporal resolution on southern toad population response to stressors in three specific ways: (1) how wetland-specific hydroregime variability and subsequent recruitment success affected long-term population dynamics, (2) how pesticide exposure during the larval stage impacted population dynamics, and (3) how chronologically specific hydroregime suitability forecasts from a climate model [30] compared to probabilistic representations of the same proportions of hydroregime classifications in different sequences.

For a standardized, realistic estimate of initial population size (not intended to be a precise measure), we created a vector comprising abundance estimates for each of the three life stages. First, we assumed upland habitat proportional to wetland area for juvenile and adult stages, estimating abundance based on overwintering density of a similar species, American toad, in enclosures (0.55 m^−2^; [36]). Then, initial proportions of pre-juveniles, juveniles, and adults were estimated based on the relative proportions of each stage for the right eigenvector of OHY matrix models, representing a stable stage distribution of approximately 81% pre-juvenile, 16% juvenile, and 3% adult. We used matrix multiplication for each year, adjusting proportion of breeders based on hydroregime classifications (OHY–0.67, MHY–0.33, IHY–0) for each wetland and year (2012–2060). A subsequent set of model projections included carbaryl effects during the larval stage and assumed a 15% reduction in larval survival [22].

Finally, we compared results of chronologically specific sequences of interannual hydroregime suitability forecasts, based on CSIRO Mk3.5 GCM projection, with probabilistic models using the same proportion of OHY, MHY, and IHY throughout the same 50-year time-period, but with randomly generated sequences. In 100 separate projections, random sequences of hydroregime suitability, and thus fecundity, were generated with the same proportion as the original classifications (e.g., 16.3% OHY, 2.0% MHY, and 81.6% IHY for wetland 7) but in a different sequence. Specific climate-based projections and randomly generated projections were compared to examine the potential uncertainty associated with predictions based on probabilistic representations of environmental stochasticity.

## 3. Results

### 3.1. Spatial Variability in Hydroregime Suitability

**Figure 2.**
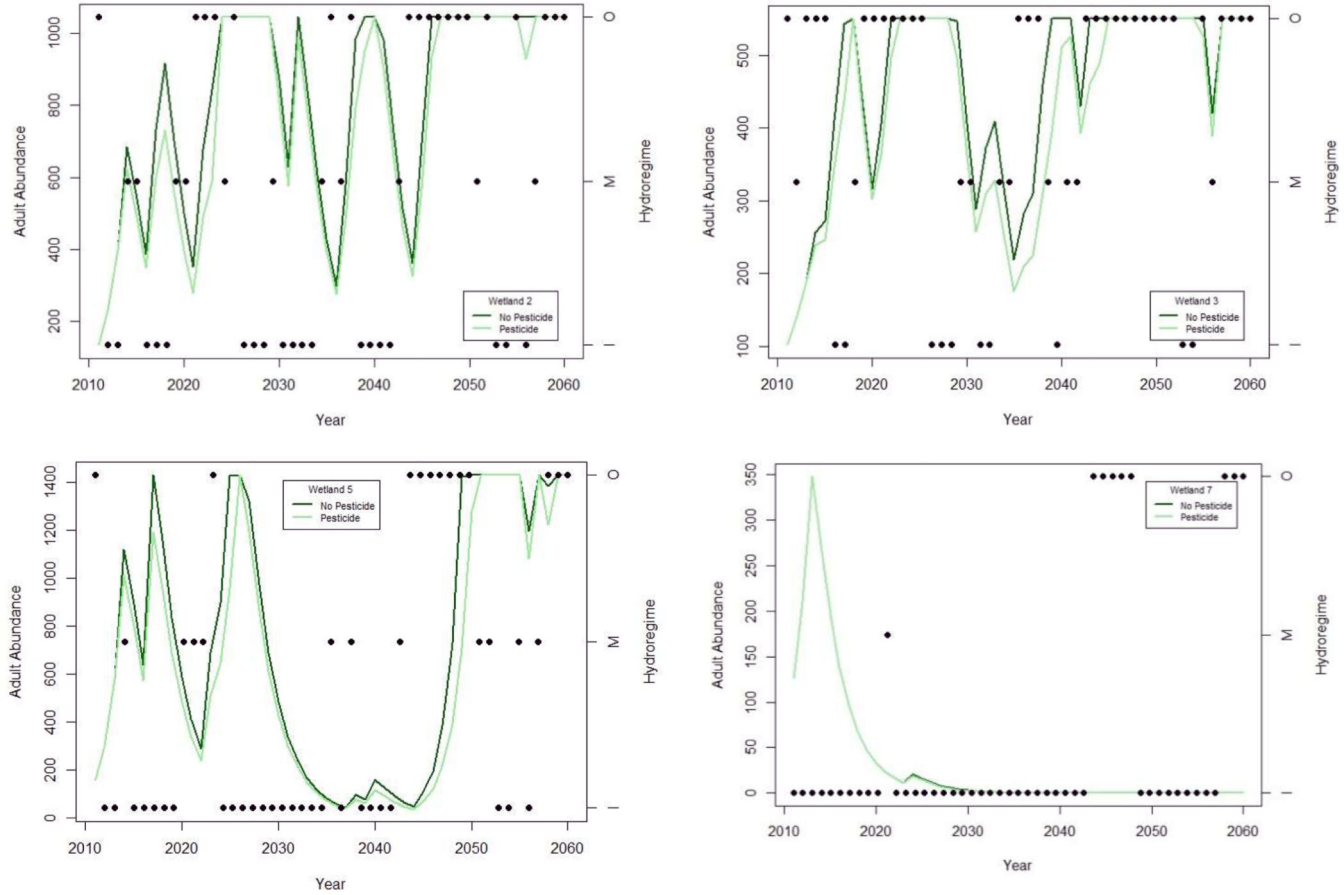
Estimated relative abundance of adults in southern toad populations, with standardized representative assumptions of density-dependent population limitation, for four patterns of hydroregime projections based on CSIRO Mk3.5 GCM climate projections at wetlands 2, 3, 5, and 7 (dark green). Population simulations varied fecundity based on hydroregime suitability of each year (OHY—points in upper plot, MHY—points in center plot, or IHY—points in lower plot) and reduced larval survival to represent aquatic exposure to carbaryl (light green).

The four wetlands modeled (2, 3, 5, 7) varied in the proportion of forecasted years having OHY (0.39, 0.59, 0.24, and 0.16 respectively), MHY (0.22, 0.20, 0.22 and 0.02), and IHY (0.39, 0.20, 0.53, and 0.82). Extended durations of IHY years dramatically reduced population size in wetlands 5 and 7 (Figure 2), with abundance approaching local extinction (<10 individuals) at wetland 5. Wetlands 2 and 3, with a higher proportion of OHY demonstrated frequently increasing population sizes that were limited by resource dependent limitations placed on them.

### 3.2. Effects of Aquatic Carbaryl Exposure

Reduced larval survival, representative of aquatic carbaryl exposure, influenced population dynamics less than the hydroregime suitability representation of fecundity (Figure 2). Population growth rates were reduced by approximately 4–6% with the inclusion of pesticide effects. This reduction impacted population fluctuations less than complete reproductive failure (IHY), which reduced population growth rates by an average of 46.6% compared with fecundity of MHY, and 57.4% compared with fecundity of OHY.

### 3.3. Probabilistic Representation of Suitable Hydroregime Frequency

**Figure 3.**
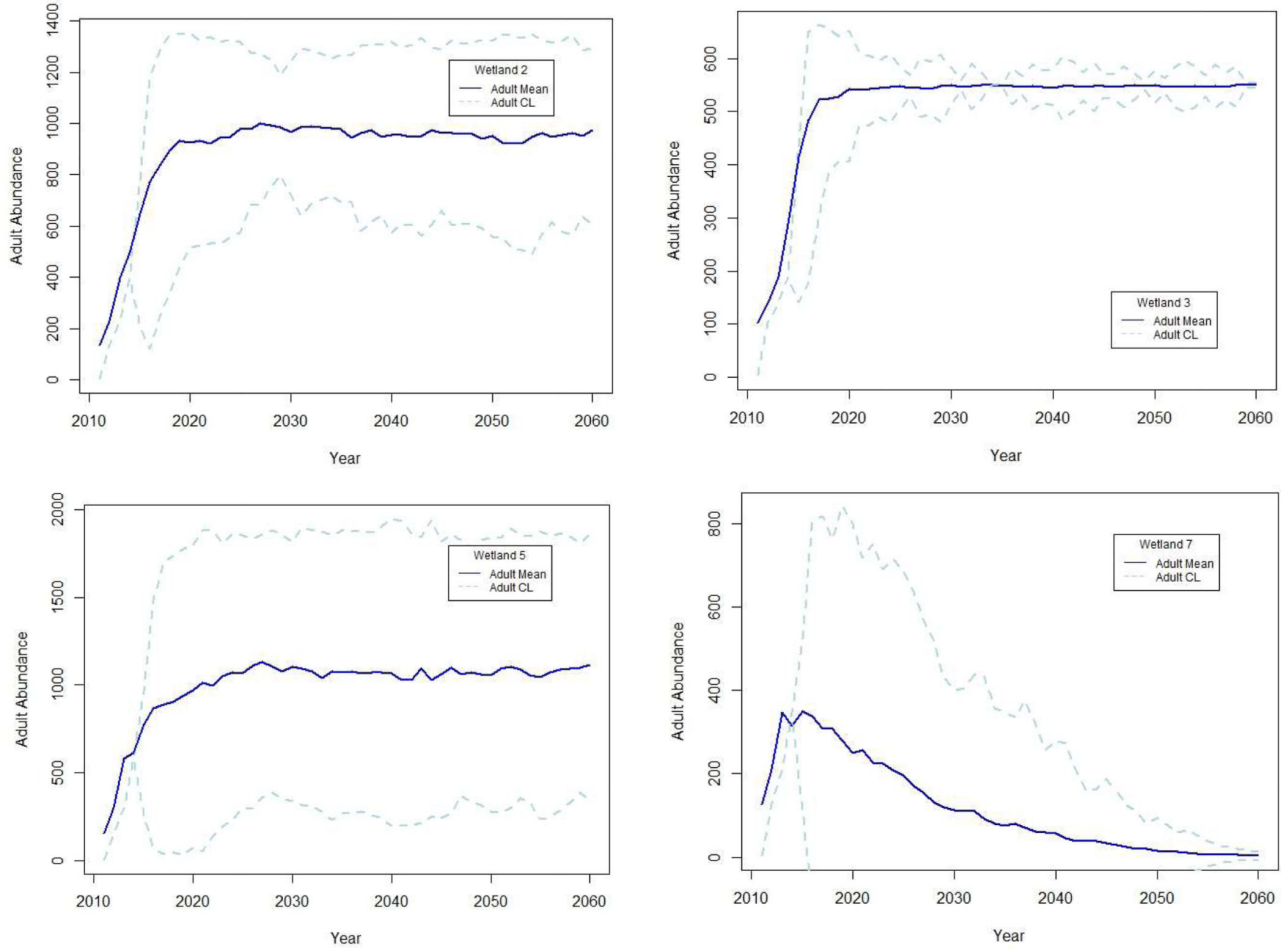
Mean and confidence limits (CL) for 100 simulated projections of estimated relative adult abundance for four patterns of hydroregimes using the same probabilities of OHY, MHY, and IHY as those predicted for wetlands 2, 3, 5, and 7 using the CSIRO Mk3.5 GCM climate projections but in no specified sequence. Probabilistic projections included the same standardized representative assumptions of density-dependent population limitation, with emphasis on environmental stochasticity effects rather than precise population estimates.

The number of consecutive years of reproductive failure influenced simulated population abundance in both chronologically specific and probabilistic models, but probabilistic models were less likely to include long sequences of any single hydroregime classification. Therefore, when the same proportion of OHY, MHY, and IHY were randomly assigned in probabilistic models, propagation of uncertainty resulted in extremely variable long-term estimates of adult abundance (Figure 3) compared to chronologically specific simulations (Figure 2). Uncertainty in probabilistic simulations was greatest in the two wetlands with relatively even distributions of hydroregime suitability classifications (wetland 2 with 0.39, 0.22, and 0.39 probability, and wetland 5 with 0.24, 0.22, and 0.53 probability of OHY, MHY, and IHY, respectively). Wetlands with probabilities more heavily skewed toward OHY (wetland 3 with 0.59) or IHY (wetland 7 with 0.82) were more precise in long-term projections of population trends, and results qualitatively corroborated the final result of simulations based on climate model forecasts of hydroregime suitability, demonstrating the same population increase (wetland 3) or decline (wetland 7; Figures 2 and 3).

## 4. Discussion

Our population projections suggested that hydroregime deficiencies predicted by the CSIRO-Mk3.5 climate model are sufficient to cause local population crashes if reproductive failure is frequent and occurs in multiple sequential years. Suitable hydroregime (OHY or MHY) in approximately 50% of years could maintain a stable population, provided that multiple successive IHYs did not occur, resulting in reduced abundance to a level of low population viability (e.g., wetland 5). Proportions of successful breeding and metamorphosis in less than 20% of the 50 years projected was insufficient to maintain stable or increasing populations in both chronologically specific and probabilistic simulations (e.g., wetland 7). Impacts of extended dry periods had a relatively greater impact on population dynamics of southern toads than aquatic pesticide exposure (Figure 2). Additionally, compensatory effects of migration from wetlands with routinely suitable hydroregime presumably would be less likely with increasing isolation of breeding populations [33,38]; however, precise migration estimates were not available for these populations.

Empirical data for amphibian populations are often complicated by low resight or recapture rates, confounding interpretation of population dynamics in the wild [6,10]). Even when demographic rates are assumed to be precise, simulations could fail to adequately describe anticipated impacts on population dynamics if stochasticity is modeled in a probabilistic way and not based on specific model-based projections of environmental variability or realistic covariance of vital rates that capture demographic variability. Alternatively, additional parameterization in probabilistic projections might capture oscillation between extended drought and rainy phases (e.g., as defined through Hidden Markov Models or similar approaches) that would, in turn, affect hydroregimes, recruitment, and population trends. Hydroregime suitability affected modeled populations in these simulations through probabilities of producing metamorphs, providing a simplistic method of differentiating a complex suite of variable environmental conditions as a categorical measure of reproductive success. Ideally, the fecundity effects of marginal hydroregime suitability should be better identified, and the criteria of this classification will vary according to duration of species’ BMP and rate of larval development. Larval development, and ultimately survival, during the sensitive transition from the aquatic stage to terrestrial habitat can impact amphibian population dynamics, especially for species with limited developmental plasticity [15]. Rapid and enduring changes in hydroregime are particularly concerning when the acceleration of pond drying exceeds the phenotypic capacity of organisms to respond to environmental cues [39].

In addition to differential response of amphibian species to environmental variability, demographic variance and minimal data present challenges in developing robust population models. Our estimation of population density and limits on terrestrial abundance reflected the paucity of empirical data available. By using a generous terrestrial density estimate and a modest assumption of upland habitat availability we were able to limit population growth in a consistent manner across wetlands. However, estimates of abundance and population growth rates are not meant to provide precise population predictions or evaluate management goals. Instead, these models manipulate parameters as a function of focal stressors (aquatic pesticide exposure and interannual hydroregime suitability for larval development) for a comparative evaluation of these impacts among wetlands. The majority of population models developed for amphibians use a similar sensitivity analysis approach rather than a precise parameterization based on detailed population data [40]. Earl [40] illustrated that population simulations for three well-studied species were robust to different assumptions about demographic variance. However, interpretation of amphibian models in the appropriate spatial and temporal context warrants significant caution, particularly when parameters are estimated or assumptions about life history, resource limitations, or multiple stressors are made for model development. A paucity of long-term field data over a range of environmental conditions hinders precise population projections for many amphibian species. Under such circumstances simulated population dynamics and sensitivity analyses are more appropriate than empirical evaluation of precise management or conservation goals.

Our simulations indicate that the temporal resolution and duration of environmental variability, and consequent outcomes for recruitment success, are relevant in assessment of amphibian population dynamics, due to their impacts on population growth rate. Although not incorporated into our southern toad models, spatial scale and metapopulation dynamics are also crucial considerations when evaluating population status, particularly for species with empirical estimates of higher dispersal and/or migration distances. Wetlands providing frequently suitable hydroregime are more likely to be a source of future breeding adults for proximate than distant wetlands with less frequent suitable hydroregimes. Additionally, determining the variability in breeding success among wetlands during MHYs is needed to reduce uncertainty in evaluation of population growth.

Evidence and projections of climate impacts on amphibian populations in the southeastern United States highlights the need for continued modeling efforts to better predict suitable hydroregimes for successful recruitment, particularly for threatened or endangered species, and in areas with anticipated increases in drought or extreme flooding [5,8,41,42]. The hydroregime suitability projections used here were based on one of many climate models available and do not represent a guaranteed prognostication of future conditions; however, the impact of reduced precipitation represented by the CSIRO Mk3.5 A1B model used here was relatively minimal in comparison with other available models (e.g., MIROC3.2 A1B; [42]). Hydroregime changes in isolated groundwater-driven ephemeral wetlands will vary regionally, depending on the timing and amount of precipitation, existing groundwater levels prior to precipitation, geomorphology, and hydrologic processes [43,44]. Decreased hydroperiod during the BMP for southern toads or any ephemeral wetland-breeding amphibian is a concern, as fewer suitable wetlands for successful recruitment could result in increased isolation and reduced inter-wetland migration within a regional metapopulation.

Our primary focus was on the importance of environmental stochasticity on hydroregime and, thus, opportunity for reproductive success. However, many other factors not accounted for in our models can affect successful recruitment. For example, we did not account for variable survival of terrestrial phase individuals or altered breeding triggers, such as rainfall or temperature, that could shift the timing of breeding periods [45], resulting in a mismatch with suitable hydroregimes during the BMP. Similarly, interactions among ecological processes and vital rates (e.g., survival, reproduction) were not considered in our models. For example, low water levels in isolated ephemeral wetlands (e.g., MHY) could result in increased density dependent larval mortality [46], accelerated tadpole development potentially resulting in smaller metamorphs and lower survival rates [47,48], increased vulnerability of eggs or larvae to predation, increased concentrations of predatory aquatic insects [49], and potentially higher concentrations of, and exposure to, contaminants. Stochastic processes affecting successful recruitment are common but poorly understood as evidenced by poor or absent correlations between breeding effort, tadpole abundance and juvenile recruitment [50] and significant, but highly variable, relationships between the number of breeding adults and recruits [10] within wetlands for multiple pond-breeding anurans. Similarly, juvenile recruitment is a significant predictor of adult populations in subsequent years for some but not all anuran species [10]. Our omission of these and other potential factors potentially affecting successful recruitment simplified our model by focusing primarily on reproductive constraints associated with environmental stochasticity and contaminants, ensuring that our projections did not overstate multiple detrimental influences.

The consistent reduction in larval survival due to carbaryl exposure modeled in each of the 50 years with sufficient hydroregime arguably over-represented the impact of pesticide use. Nonetheless, the comparative effects of continuous aquatic pesticide exposure were substantially less detrimental than reproductive failure associated with unsuitable hydroregimes in these representations. The comparative references to population growth rates provide a relative perspective on environmental and extrinsic impacts for the “general and realistic” goals of our designated risk assessment approach and are not intended to be precise management criteria. Similarly, density-dependent constraints simulated in our models were implemented to limit population growth, and specific values of population size should not be inferred as precise estimations of abundance at these wetlands.

Our goal was to project a generally applicable, realistic scenario of suitable hydroregimes for successful recruitment by a common, representative amphibian species breeding in ephemeral wetlands, and to simulate future population dynamics. These simulations suggest a relatively low-risk scenario for our common focal species. However, further ramifications of reduced hydroregime suitability and altered environmental conditions might be considered for species with limited distribution or dispersal, prolonged rates of larval development, or less plastic life history strategies. Additional modeling parameters should be considered for more specific habitat dependencies (e.g., upland matrices or wetland characteristics) as well as additional demographic or ecological constraints that would better represent metapopulation dynamics.

In general, the PopGUIDE framework offers a functional, stepwise method of constructing a basic population model, in this case to examine relative population sensitivities; however, the constraints of both data availability and representative temporal and spatial scale, particularly for anurans dependent on ephemeral wetlands, should be considered when compiling data and parameterizing a model. The sensitivity approach is a common strategy to obtain some perspective on population dynamics [40], but specific management goals (e.g., for small populations or threatened species) will rely on precise demographic data and spatial and temporal detail that is often not accessible, suggesting prudence in interpretation of abundance estimates or deterministic model results. An interactive modeling tool that allowed inclusion of parameter uncertainty or flexibility in defining spatial structure and/or temporal scale could provide a useful preliminary evaluation of population dynamics where specific estimates are lacking.

## Figures and Tables

**Table 1.** Data compilation for southern toad model.

	Characteristic	Estimate	Source
Organism-level characteristics	Life span Reproductive season Reproductive frequency Clutch size Onset of maturation Embryo-larval survival Metamorph survival Juvenile survival Immature transition rate Sex ratio Adult survival	10 years mid-February to mid-August hydroregime dependent 3829 first year, 4431 adult years 3 years, female 0.012 0.3 0.3 0.1 0.5 0.7	AnAge database [30] [30] [14] AnAge database; [14] Based on [14] [14] Based on [14] Based on [14] assumed even Based on [14]
Population and spatial characteristics	Density dependence Population size Spatial structure Movement Habitat features Geographical range Hydroregime suitability	in larval development, terrestrial capacity wetland-specific density small breeding wetlands with minimal migration low percentage of migration between close wetlands eight ephemeral breeding wetlands US southeast hydroregime sufficient for larval survival	[31,35] [30] [30] [6] [30] [30] [30]
Extrinsic factors	Predation Competition Environmental conditions Pathogens Abiotic stressors Management Indirect effects Stochasticity	no fish predation in temporary wetlands; no aquatic invertebrate predation included here not evident highly variable hydroregime many; not identified in breeding range habitat degradation; not identified in study area not included in model not included in model environmental	[30] assumption [30] assumption assumption NA NA [30]
Exposure and effects characterization	Chemical exposure Temporal exposure Exposure across habitat Toxic effects Effects by life stage Effects by exposure route	carbaryl exposure larval stage aquatic habitat reduced survival reduced larval survival not specified	simulation assumption simulation assumption [20] NA

## Data Availability

Not applicable.
